# Chronic complications and quality of life of patients living with sickle cell disease and receiving care in three hospitals in Cameroon: a cross-sectional study

**DOI:** 10.1186/s12878-017-0079-7

**Published:** 2017-04-20

**Authors:** Anne M. Andong, Eveline D. T. Ngouadjeu, Cavin E. Bekolo, Vincent S. Verla, Daniel Nebongo, Yannick Mboue-Djieka, Simeon-Pierre Choukem

**Affiliations:** 1 Department of Internal Medicine and Pediatrics, Faculty of Health Sciences, University of Buea, Buea, Cameroon; 2 Health and Human Development (2HD) Research Network, P.O. Box 4856, Douala, Cameroon; 3 Faculty of Medicine and Pharmaceutical Sciences, University of Douala, Douala, Cameroon; 4 Department of Internal Medicine, Douala General Hospital, P.O. Box 4856, Douala, Cameroon; 5 Ministry of Public Health, Centre Medical d’Arrondissement de Bare, Nkongsamba, Cameroon

**Keywords:** Sickle cell disease, Chronic complications, Prevalence, Quality of life, Cameroon

## Abstract

**Background:**

Sickle Cell Disease (SCD) is associated with chronic multisystem complications that significantly influence the quality of life (QOL) of patients early in their life. Although sub-Saharan Africa bears 75% of the global burden of SCD, there is a paucity of data on these complications and their effects on the QOL. We aimed to record these chronic complications, to estimate the QOL, and to identify the corresponding risk factors in patients with SCD receiving care in three hospitals in Cameroon.

**Methods:**

In this cross-sectional study, a questionnaire was used to collect data from consecutive consenting patients. Information recorded included data on the yearly frequency of painful crisis, the types of SCD, and the occurrence of chronic complications. A 36-Item Short Form (SF-36) standard questionnaire that examines the level of physical and mental well-being, was administered to all eligible participants. Data were analyzed with STATA® software.

**Results:**

Of 175 participants included, 93 (53.1%) were female and 111 (aged ≥14 years) were eligible for QOL assessment. The median (interquartile range, IQR) age at diagnosis was 4.0 (2.0-8.0) years and the median (IQR) number of yearly painful crisis was 3.0 (1.0–7.0). The most frequent chronic complications reported were: nocturnal enuresis, chronic leg ulcers, osteomyelitis and priapism (30.9%, 24.6%, 19.4%, and 18.3% respectively). The prevalence of stroke and avascular necrosis of the hip were 8.0% and 13.1% respectively. The median (IQR) physical and mental scores were 47.3 (43.9–58.5) and 41.0 (38.8–44.6) respectively. Age and chronic complications such as stroke and avascular necrosis were independently associated with poor QOL.

**Conclusions:**

In this population of patients living with SCD, chronic complications are frequent and their QOL is consequently poor. Our results highlight the need for national guidelines for SCD control, which should include new-born screening programs and strategies to prevent chronic complications.

**Electronic supplementary material:**

The online version of this article (doi:10.1186/s12878-017-0079-7) contains supplementary material, which is available to authorized users.

## Background

Sickle cell disease (SCD) is often associated with chronic complications in the long term [1]. These complications include consequences of chronic anemia and susceptibility to infections owing to functional splenectomy, and may lead to a poor quality of life (QOL) [2]. The World Health Organization (WHO) estimates that 300,000 children are born with SCD each year, 75% of whom are in sub-Saharan Africa (SSA); they also state that the burden of the disease could be reduced by simple careful management and prevention programs [3, 4].

The WHO also recommends that a global management be put in place to reduce SCD morbidity (chronic complications) and mortality, and to improve on the QOL [3]. The disease morbidity and mortality improved in two small samples of patients in Nigeria and Angola using these simple but cost-effective interventions recommended by the WHO [5, 6]. However, data especially those focusing on QOL are still scanty or inexistent in most SSA countries.

To the best of our knowledge, chronic complications that have been reported in Cameroon –a SCD endemic country- are stroke (6.7%) [7] and gall stone diseases (30%) [8]. The aim our study was to assess a group of children and adults living with SCD, with particular reference to the types and determinants of the QOL and complications of SCD, in order to inform the actions that could be undertaken to reduce the burden of the disease and to improve the QOL.

## Methods

### Study design, setting and participants

We carried out a cross-sectional study over a period of five months, from November 2014 to March 2015, in three hospitals in Cameroon: the Douala General Hospital, a country reference hospital, the Douala Laquintinie Hospital, which has a sickle cell care center in Douala, and the Buea Regional Hospital, which is the reference hospital for the South-West Region of the country. The first two hospitals are located in the Littoral Region and the third in South-West Region. The two regions are at the coastal area of the country and serve as university teaching hospitals for our Faculty of Health Sciences of The University of Buea. Recruitment of participants was done at the inpatient and outpatient units and during their monthly visits at the Laquintinie Hospital. All the centers had comprehensive medical records. All patients with SCD confirmed by a hemoglobin electrophoresis were consecutively included if they gave their informed written consent or assent to participate. Patients in sickle cell crises at the time of the study were not considered (for QOL assessment), until they completely recovered from the crises as indicated by their caring physician.

All patients above the age of 5 years were included in the assessment of chronic complications because complications such as stroke have been shown to occur as early as the age of 5 years in children with sickle cell disease [9]. Only patients above 14 years were included in the assessment of the QOL, because the short form 36 questionnaire has been validated in sickle cell population at or above this age [10].

### Definition of terms and variables

Sickle cell disease had been diagnosed by haemoglobin electrophoresis. Painful sickle cell crises were defined as any bony painful event in the absence of any recent trauma, for which a medical consultation was done or not. Chronic leg ulcers were defined as any leg wound that had lasted longer than three months. Stroke was defined as any sudden onset neurological dysfunction (mainly one side body weakness) in the absence of trauma, that resolved or not. Avascular necrosis of the hip was self-reported or collected from medical records. Immunization status relating to pneumococcal vaccine, meningococcal vaccine, Typhim Vi (against typhoid fever) and Hepatitis B vaccine) was also recorded.

### Data collection and score calculation

All participants underwent a comprehensive multisystem physical examination. Data on chronic complications were collected by use of a questionnaire (Additional file 1) that contained information on the history obtained from the participant and/or the guardian. Additional clinical data were retrieved from the patient’s medical record.

The Short Form (SF)-36 either self-administered or interviewer-administered was used to collect data on the QOL (Additional file 1). This questionnaire reviewed both physical and mental aspect of health comprising eight scored scales each belonging to the two main scores known as Physical and Mental health component scores. The SF 36 form was clearly explained and self-administered except for participants who did not understand the questions. All the 8 components were assessed through 11 questions. An overall score, physical component score and mental component score were computed. Individual scores for all 8 components were also given. The scores were then standardized using the online newborn screening (NBS) calculator so that the values could be compared to other populations, both the healthy and sickle cell populations. Only one investigator administered the questionnaire to all participants.

### Data analysis

The data set was checked for logical inconsistencies, invalid codes, omissions and improbable data by tabulating, summarizing, describing and plotting variables, depending on their nature. Missing observations were systematically excluded. Summary statistics were presented as proportions for categorical variables, as mean and standard deviation for normally distributed continuous variables and as median and interquartile range (IQR) for continuous variables with a skewed distribution. Associations between QOL scores and exposure variables were evaluated by a linear regression model. Variables associated with Total SF-36 score, PCS and MCS in separate univariate analyses at the 5% significance level were included in respective multivariate linear regression models. Backward elimination based on a p-value lower than 0.05 was used to retain variables that were independently associated with each QOL scores. Adjusted regression and correlation coefficients with their p-values and 95% confidence intervals (CI) were obtained. The goodness of model fit was assessed by post-estimation of homoscedasticity of residuals.

## Results

**Fig. 1:**
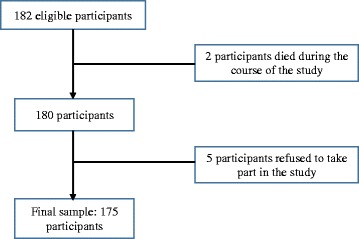
Flow chart of the inclusion process

In this study 182 participants met the inclusion criteria of which 175 finally took part in the study giving a response rate of 96.2% (Fig. 1). Of these, 94 (54.9%) were recruited at Laquintinie Hospital, 72 (41.1%) at Douala General Hospital and 9 (5.1%) at Buea Regional Hospital.

### General characteristics of the participants

Of the 175 participants, 93 (53.1%) were females. The median (IQR) age at which the diagnosis of SCD was confirmed was 4.0 years (2.0–8.5); 113 (64.6%) participants were below the age of 21 and therefore needed consent by proxy, 21 being the age of majority in Cameroon. The median (IQR) age was 16.0 years (9.0–24.0). Concerning ongoing treatment, 122 (69.7%) patients were on folic acid alone, 1 (0.6%) on a vasodilator only, 17 (9.7%) on both folic acid and hydroxyurea, 21 (12.0%) on both folic acid and a vasodilator, 1 (0.6%) were on all three medications and 13 (7.4%) were not taking any medication. Most of the participants 103 (58.9%) did not have their vaccines up-to-date. Only 75(42.9%) had regular medical follow-up at the time of the study, 45(25.7%) being seen by pediatricians. Hemoglobin electrophoresis results were available for 58 (33.1%) participants and distributed as follow: 28 (48.3%) SSFA_2_, 16 (27.6%) SSA_2_, 10 (17.2%) SSF, 3 (5.2%) SS, and 1 (1.7%) SSC. The median number of yearly painful sickle cell crisis was 3.0 (IQR: 1.0–7.0). Details of other characteristics are shown in Table 1.

**Table 1:** General characteristics of the study participants

Characteristics		Number	Percent
Gender	Females	93	53.1
	Males	82	46.9
Age (years)	5–15	81	46.3
	≥16	94	53.7
Education	None	4	2.3
	Primary	65	37.1
	Secondary	73	41.7
	Tertiary	33	18.9
Marital status	Single	168	96.0
	Married	7	4.0
Residence	Rural	9	5.1
	Urban	166	94.9
Employment Status	Unemployed	155	88.6
	Employed	19	10.9

### Chronic complications

Details of chronic complications found in participants are depicted in Table 2. The mean (SD) age at first stroke was 12.6 (7.3) years. The majority (52.2%) of cases of avascular necrosis were on the right hip. The mean age at which participants had their first leg ulcer was 18.6 ± 7.9 years. Forty (22.9%) patients had a systolic murmur. The prevalence of enuresis was similar between children and adults. Eight (4.6%) participants had opioid tolerance.

**Table 2:** Chronic complications of sickle cell disease

Complications	Number (*n*)	Prevalence (%)
Ischemic complications		
Refractive eye disorders	47	26.9
Avascular necrosis of the hip	23	13.1
Priapism	15	18.3^a^
Stroke	14	8.0
Anemic complications		
Heart disease	46	26.2
*Chronic leg ulcers*	43	24.6
*Gall stones*	12	6.9
Infectious complications		
*Osteomyelitis*	34	19.4
*Septic arthritis*	24	13.7
*Tuberculosis*	9	5.1
*Others*		
*Enuresis*	54	30.9
*Sleep apnoea*	10	5.4

^a^The denominator included males only (*n* = 82)

### Quality of life

**Fig. 2:**
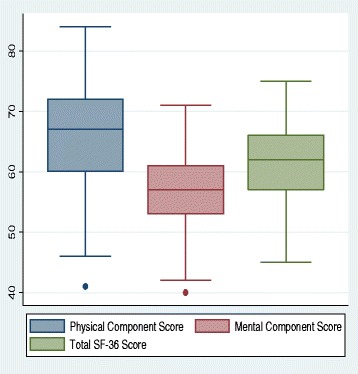
Box plot summarizing quality of life scores. Blue: physical component scores; Red mental component scores; Green: total SF-36 scores. Isolated points are outliers

**Fig. 3:**
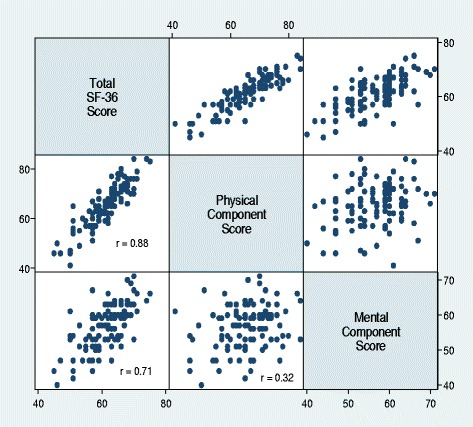
Correlation matrix between the SF-36 components of quality of life score. The squares with scattered plots represent the areas and directions of correlation of the total scores and the specific scores

All 111 participants aged 14 years and above were included in the QOL study; their median (IQR) physical component score (PCS) and mental component score (MCS) were 47.3 (43.9–58.5) and 41.0 (38.8–44.6), respectively. The median (IQR) total SF-36 score was 62 (57–66) (Fig. 2). The total score strongly correlated with both the PCS (*r* = 0.88, *p* = 0.01) and the MCS (0.71, *p* = 0.04) (Fig. 3).

Independent associations of QOL scores are shown in Table 3. Holding everything else constant, on average: every one year increase in age was associated with a decrease in the total QOL score by 0.15 point; urban dwellers had a total score that was about 8 points above that of patients living in rural areas; stroke was associated with about a 5-point reduction in the total score while ANH was associated with a 3-point reduction in the total QOL score. Factors associated with poor PCS were ageing, stroke, ANH and chronic leg ulcer. The MCS was higher in urban residents but was lower in females and in patients who had gall stones. Other coefficients were not significantly different from zero.

**Table 3:** Multiple Linear regression models of factors associated with quality of life scores

*Factor*	*Regression coefficient (ß)*	*95% confidence interval*	*P-value*
Total SF-36 Score, F(4, 105)			
Age	−0.15	−0.28 to −0.02	0.025
Urban residence	7.92	2.86 to 13.00	0.002
Stroke	−4.85	−8.76 to −0.94	0.015
Avascular necrosis of the hip	−3.35	−6.11 to −0.60	0.018
Physical Component Score, F(4, 105)			
Age	−0.25	−0.43 to −0.07	0.007
Stroke	−8.68	−14.12 to −3.24	0.002
Avascular necrosis of the hip	−4.63	−8.45 to −0.80	0.018
Chronic leg ulcer	−3.70	−6.75 to −0.65	0.018
Mental Component Score, F(4, 105)			
Females	−2.46	−4.78 to −0.14	0.038
Urban residence	8.29	2.76 to 13.80	0.004
Gall stones	−4.02	−7.70 to −0.34	0.033

*SF* Short form, *F* regression analysis significance

## Discussion

We found in this study that our participants living with SCD are diagnosed late (median of 4 years) contrary to WHO recommendations of new-born screening and diagnosis. With a median age of 16 years, they presented frequent and multiple chronic complications of which 8% of stroke. The level of care is globally below the standards, and consequently the QOL is poor with the severity associated with age and the presence of chronic complications.

Though late, the diagnosis was however done earlier than was reported by Wonkam et al. in Yaoundé (8.3 years), the capital of Cameroon, in 2014 [11]. Countries that have instituted newborn screening such as Belgium have their diagnosis done earlier (median 0.7 years) have improved the mortality and morbidity of the disease and therefore a better quality of life [12]. Late diagnosis in Cameroon potentially affects the health status and quality of life of the individual, as well as the whole family’s economic and psychological wellbeing.

The participants of this study had more frequent painful crises per year than that reported in nearby Nigeria [13] which has adopted systematic follow-up of patients. In addition, only 57% of our sickle cell population have had some kind of medical follow-up compared to SCD populations in the USA where more than 9 out of 10 do have regular follow up [14].

The prevalence of stroke in this study (8.0%), was similar to that reported by Njamnshi et al. in 2006 in Yaoundé (6.67%) [7]. About two fifth of our male study population suffered from priapism (18.3%), compared to other sickle cell populations in Africa like in Nigeria, where the prevalence was 39.1% in adults [15]. This probably reflects differences in the age groups studied, as we found that increasing age was significantly associated with priapism. The same reason may explain the lower prevalence of avascular necrosis (AVN) of the hip in our study (13.1%) compared with reports from the USA in 2014 (29%) [16].

The high prevalence of leg ulcers (24.6%) is a good indicator of lack of medical follow-up in our study population [17, 18]. The prevalence of osteomyelitis in this study (19.4%) was similar to that obtained from other sickle cell populations [19]. Nocturnal enuresis was the chronic complication with the highest prevalence affecting about one in three persons of our study population (30.1%). This prevalence was comparable to what has been reported from other studies [20, 21].

All median SF-36 scores in our study were lower than the USA norms studied and implemented in 1998 [10]. This indicates a globally low QOL in our population. The median PCS and MCS we report here (47.3 and 41.0 respectively) suggest that our study population had below normal physical and mental health.

Considering the study of Abdel-Monhem Amr et al. conducted in Saudi Arabia in 2011, all the median SF-36 scores in our study were higher than those of their sickle cell population but lower than that of their healthy population [22]. They used a narrower age group (14–18 years) and their questionnaire was administered even during painful episodes.

Our study has potential limitations. Being conducted in the hospital rather than the community, we have probably lost some information like lifestyle factors which can influence patients’ outcome. Though all patients had had a confirmed diagnosis of SCD by electrophoresis, we could access only 33% of results, which did not allow a strong assessment of a potential association between genotype and QOL. Also, self-reporting of chronic complications may have caused potential report bias. Finally, some files did not have all information required. However, we have provided accurate data on the current standards of care and QOL of individuals living with SCD that may probably guide stakeholders to improve on policies on screening and follow up of these patients in Cameroon.

## Conclusions

The prevalence of chronic complications in sickle cell patients in Cameroon is higher than in most other Sickle cell populations worldwide, probably due to late diagnosis. Chronic complications are also common and are the main drivers of low QOL. Our results highlight the need for national guidelines for SCD control, which should include early or newborn screening programs.

## Additional file

Additional file 1: Questionnaire. (DOCX 21 kb)

## Abbreviations

ANH: Avascular necrosis of the hip
IQR: Interquartile range
MCS: Mental component score
NBS: New-born screening
PCS: Physical component score
QOL: Quality of life
SCA: Sickle cell anemia
SCD: Sickle cell disease
SD: Standard deviation
SF-36: Short Form-36
WHO: World Health Organization
