# Supplementary material for: Chronic complications and quality of life of patients living with sickle cell disease and receiving care in three hospitals in Cameroon: a cross-sectional study
Source: BMC Hematol. 2017 Apr 20;17:7. doi: 10.1186/s12878-017-0079-7 (PMC5399423; doi:10.1186/s12878-017-0079-7)
Supplement: Additional file 1: — Questionnaire. (DOCX 21 kb) [file 12878_2017_79_MOESM1_ESM.docx]

**QUESTIONNAIRE**

**Identification code …………..**

**PART ONE- SOCIO DEMOGRAPHIC DATA**

1. **Date of birth /__/__//__/__//__/__/__/__/**
2. **Sex:** male (m) /female (f) **……………………**
3. **Highest level of education; ………………..** 0=none 1=pre-primary 2=primary 3=secondary 4=skills training 5=tertiary
4. **Employment status; ………………..** 0=unemployed 1=employed 2=retired
5. **Occupation** ……………………………………………………………
6. **Marital status ……………... 0**=single 1=married 2=widow/widower 3=divorced
7. **Residence** …………………………………………….

**PART TWO: QUESTIONNAIRE TO ASSESS CHRONIC COMPLICATIONS**

Write the correct answer in the space provided.

1. Tick if you have or have ever had any of the following. This will be completed with the help of the investigator. For each, please record the year(y) and number of episodes(n).

| Stroke | Leg ulcer for ≥3 months | Pulmonary TB | Sleep apnoea |
| --- | --- | --- | --- |
| y; |  |  |  |
|  | y; | y; | y; |
| n; | n; | n; | n; |
| Sustained painful erection | cardiomyopathy | osteomyelitis | enuresis |
| y; | y; | y; | y; |
| n; | n; | n; | n; |
| Chronic Limb due to hip pain |  | Septic arthritis | Opioid tolerance |
| y; |  | y; | y; |
| n; |  | n; | n; |
| kidney (include ESRD, take BP) |  |  | Pulmonary hypertension |
| y; |  |  |  |
|  |  |  | y; |
| n; |  |  | n; |
| Corrective glasses |  |  | Others; |
| y; |  |  | y; |
| n; |  |  | n; |
| Gall stone disease |  |  |  |
| y; |  |  |  |
| n; |  |  |  |

1. How old were you when you were diagnosed of sickle cell anaemia by an electrophoresis? ………………
2. What was the electrophoresis profile and their percentages? (10 and 11 for investigator)

| Hb | Hb S | Hb A | Hb F | Hb A2 | Hb A1 | Hb C | others |
| --- | --- | --- | --- | --- | --- | --- | --- |
| % |  |  |  |  |  |  |  |

1. Type of sickle cell (e.g. SF SAF, SAFA etc. from combining the above) ……………...
2. How many crises have you had over the last 12 months? ……………..
3. Out of the crises above, how many were managed in the hospital? ……………
4. Which drugs do you take regularly and how often for each? ……………………………

……………………………………………………………………………………………………………………………………………………………………………………………………

1. How many transfusions have you had in the last 12 months? ………………
2. Tick if you have any of these other diseases.

| G6PD deficiency | Hepatitis B | Hepatitis C | HIV/AIDS | Asthma | Others (specify) |
| --- | --- | --- | --- | --- | --- |
|  |  |  |  |  |  |

1. Are you being followed up? Yes/No ………………
2. If Yes, for how long? ………………
3. If yes in 17 above, by who? Tick below

| Haematologist | General practitioner | Nurse | Others (specify) |
| --- | --- | --- | --- |
|  |  |  |  |

20) If yes in 17 above: how is your state of health since then? Better/worse/no difference? ...................................

**PART THREE: QUALITY OF LIFE SCALE**

**SF-36 Health Survey for quality of life assessment**

**Study number: ……………… Date when completing form: ……………**

**INSTRUCTIONS:** This survey asks your views about your health. This information will help keep track of how you feel and how well you are able to do your usual activities. Please answer every question by marking the answer as indicated. If you are unsure about how to answer a question, please give the best answer you can.

When complete, please return the questionnaire.

| 1. In general, would you say your health is: (Circle one)   Excellent………..…………………………………………………………….. 1  Very good .……………………………………………………………………. 2  Good .……………………………………………………………………..……3  Fair ………………………………………………………………………….... 4  Poor …………………………………………………………………………... 5   1. Compared to one year ago, how would you rate your health in general now? (Circle one)   Much better now than one year ago ………………………………………………………. 1  Somewhat better than one year ago ………………………………………………………. 2  About the same as one year ago ………………………………………………………….. 3  Somewhat worse than one year ago ……………………………………………………… 4  Much worse now than one year ago ……………………………………………………… 5   1. The following questions are about activities you might do during a typical day. Does your health now limit you in these activities? If so, how much?   (circle one number on each line)   \|  \| Yes, limited  a lot \| Yes, limited a  little \| No, not  limited at all \| \| --- \| --- \| --- \| --- \| \| 1. Activities \| 1 \| 2 \| 3 \| \| 1. Vigorous activities, such as running, lifting heavy objects, participating in strenuous sports. \| 1 \| 2 \| 3 \| \| 1. Moderate activities, such as moving a table, \| 1 \| 2 \| 3 \| \| 1. Lifting or carrying groceries \| 1 \| 2 \| 3 \| \| 1. Climbing several flights of stairs \| 1 \| 2 \| 3 \| \| 1. Climbing one flight of stairs \| 1 \| 2 \| 3 \| \| 1. Bending, kneeling or stooping \| 1 \| 2 \| 3 \| \| 1. Walking more than a kilometre \| 1 \| 2 \| 3 \| \| 1. Walking half a kilometre \| 1 \| 2 \| 3 \| \| 1. Walking one hundred metres \| 1 \| 2 \| 3 \| \| 1. Bathing or dressing yourself \| 1 \| 2 \| 3 \|   4. During the past 4 weeks, have you had any of the following problems with your work or other regular daily activities as a result of your physical health?  (Circle one number on each line)   \| Yes \| No \| \| --- \| --- \| \| 1. Cut down on the amount of time you spent on work or other activities \| 1 \| 2 \| \| \| 1. Accomplished less than you would like \| 1 \| 2 \| \| \| 1. Were limited in the kind of work or other activities \| 1 \| 2 \| \| \| 1. Had difficulty performing the work or other activities (for example, it took extra effort) \| 1 \| 2 \| \|   5. During the past 4 weeks, have you had any of the following problems with your work or other regular daily activities as a result of any emotional problems (such as feeling depressed or anxious)?  (Circle one number on each line)   \|  \| Yes \| No \| \| --- \| --- \| --- \| \| 1. Cut down on the amount of time you spent on work or other activities \| 1 \| 2 \| \| 1. Accomplished less than you would like \| 1 \| 2 \| \| 1. Didn’t do work or other activities as carefully as usual \| 1 \| 2 \|   6. During the past 4 weeks, to what extent has your physical health or emotional problems interfered with your normal social activities with family, friends, neighbours or groups?  (circle one)  Not at all …………………………………………………………………………………… 1  Slightly …………………………………………………………………………………….. 2  Moderately ………………………………………………………………………………… 3  Quite a bit …………………………………………………………………………………..4  Extremely ………………………………………………………………………………….. 5  7. How much bodily pain have you had during the past 4 weeks? (circle one)  None ……………………………………………………………………………………..……1  Very mild ……………………………………………………………………………………. 2  Mild …………………………………………………………………………………………. 3  Moderate ……………………………………………………………………………………. 4  Severe ……………………………………………………………………………………….. 5  Very severe ………………………………………………………………………………… 6  8. During the past 4 weeks, how much did pain interfere with your normal work (including both work outside the home and housework) (circle one)  Not at all ………………………………………………………………………………….. 1  A little bit ………………………………………………………………………………….. 2  Moderately ………………………………………………………………………………….3  Quite a bit ………………………………………………………………………………….. 4  Extremely ………………………………………………………………………………….. 5  9. These questions are about how you feel and how things have been with you during the past 4 weeks. For each question please give the one answer that comes closest to the way you have been feeling.How much of the time during the past 4 weeks…   \|  \| All of  the time \| Most of the  time \| A good bit  of the time \| Some of  the time \| A little of  the time \| None of the  time \| \| --- \| --- \| --- \| --- \| --- \| --- \| --- \| \| 1. Did you feel full of life \| 1 \| 2 \| 3 \| 4 \| 5 \| 6 \| \| 1. Have you been a very nervous person? \| 1 \| 2 \| 3 \| 4 \| 5 \| 6 \| \| 1. Have you felt so down in the dumps that nothing could cheer you up? \| 1 \| 2 \| 3 \| 4 \| 5 \| 6 \| \| 1. Have you felt calm and peaceful? \| 1 \| 2 \| 3 \| 4 \| 5 \| 6 \| \| 1. Did you have a lot of energy? \| 1 \| 2 \| 3 \| 4 \| 5 \| 6 \| \| 1. Have you felt downhearted and low? \| 1 \| 2 \| 3 \| 4 \| 5 \| 6 \| \| 1. Did you feel worn out? \| 1 \| 2 \| 3 \| 4 \| 5 \| 6 \| \| 1. Have you been a happy person? \| 1 \| 2 \| 3 \| 4 \| 5 \| 6 \| \| 1. Did you feel tired? \| 1 \| 2 \| 3 \| 4 \| 5 \| 6 \|   10. During the past 4 weeks, how much of the time has your physical health or emotional problems interfered with your social activities (like visiting friends, relatives, etc.)?  (circle one)  All of the time …………………………………………………………………………………… 1  Most of the time …………………………………………………………………………………. 2  Some of the time ………………………………………………………………………………….3  A little of the time ……………………………………………………………………………….. 4  None of the time …………………………………………………………………………………. 5  11. How TRUE or FALSE is each of the following statements to you?  (circle one number on each line)   \|  \| Definitely true \| Mostly true \| Don’t know \| Mostly false \| Definitely false \| \| --- \| --- \| --- \| --- \| --- \| --- \| \| 1. I seem to get ill more easily than other people \| 1 \| 2 \| 3 \| 4 \| 5 \| \| 1. I am as healthy as anybody I know \| 1 \| 2 \| 3 \| 4 \| 5 \| \| 1. I expect my health to get worse \| 1 \| 2 \| 3 \| 4 \| 5 \| \| 1. My health is excellent \| 1 \| 2 \| 3 \| 4 \| 5 \| |
| --- | --- | --- | --- | --- | --- | --- | --- | --- | --- | --- | --- | --- | --- | --- | --- | --- | --- | --- | --- | --- | --- | --- | --- | --- | --- | --- | --- | --- | --- | --- | --- | --- | --- | --- | --- | --- | --- | --- | --- | --- | --- | --- | --- | --- | --- | --- | --- | --- | --- | --- | --- | --- | --- | --- | --- | --- | --- | --- | --- | --- | --- | --- | --- | --- | --- | --- | --- | --- | --- | --- | --- | --- | --- | --- | --- | --- | --- | --- | --- | --- | --- | --- | --- | --- | --- | --- | --- | --- | --- | --- | --- | --- | --- | --- | --- | --- | --- | --- | --- | --- | --- | --- | --- | --- | --- | --- | --- | --- | --- | --- | --- | --- | --- | --- | --- | --- | --- | --- | --- | --- | --- | --- | --- | --- | --- | --- | --- | --- | --- | --- | --- | --- | --- | --- | --- | --- | --- | --- | --- | --- | --- | --- | --- | --- | --- | --- | --- | --- | --- | --- | --- | --- | --- | --- | --- | --- | --- | --- | --- | --- | --- | --- | --- | --- | --- | --- | --- | --- | --- | --- | --- | --- | --- | --- | --- | --- | --- | --- |
